# Application of Surface-Enhanced Raman Spectroscopy Combined with Immunoassay for the Detection of Adrenoceptor Agonists

**DOI:** 10.3390/foods13121805

**Published:** 2024-06-08

**Authors:** Yao Wang, Yubing Jing, Jinbo Cao, Yingying Sun, Kaitong Guo, Xiujin Chen, Zhaozhou Li, Qiaoqiao Shi, Xiaofei Hu

**Affiliations:** 1 Henan International Joint Laboratory of Food Green Processing and Quality Safety Control, College of Food and Bioengineering, Henan University of Science and Technology, Luoyang 471023, China; 210321090624@stu.haust.edu.cn (Y.J.); cjinbo2024@163.com (J.C.); 210321090620@stu.haust.edu.cn (Y.S.); 210321090667@stu.haust.edu.cn (K.G.); chenxiujin9610@haust.edu.cn (X.C.); lizhaozhou@haust.edu.cn (Z.L.); 2 Henan Key Laboratory of Animal Immunology, Henan Academy of Agricultural Sciences, Zhengzhou 450002, China; 3 School of Grain Science and Technology, Jiangsu University of Science and Technology, Zhenjiang 212100, China; qqshi2018@just.edu.cn

**Keywords:** adrenoceptor agonists, SERS, immunoassays, substrate materials, Raman reporter molecules

## Abstract

Rapid, sensitive, and accurate detection of adrenoceptor agonists is a significant research topic in the fields of food safety and public health. Immunoassays are among the most widely used methods for detecting adrenoceptor agonists. In recent years, surface-enhanced Raman spectroscopy combined with immunoassay (SERS-IA) has become an effective technique for improving detection sensitivity. This review focuses on the innovation of Raman reporter molecules and substrate materials for the SERS-IA of adrenoceptor agonists. In addition, it also investigates the challenges involved in potentially applying SERS-IA in the detection of adrenoceptor agonists. Overall, this review provides insight into the design and application of SERS-IA for the detection of adrenoceptor agonists, which is critical for animal-derived food safety and public health.

## 1. Introduction

Adrenoceptor agonists, as sympathomimetic amines, primarily exhibit pharmacological effects through cardiovascular stimulation, bronchial smooth muscle inhibition, and metabolic promotion. These medications are authorized for human medical purposes. Some animal studies have shown that adrenoceptor agonists can enhance muscle protein synthesis and expedite fat conversion and breakdown; therefore, many farms use these agonists as animal feed additives [1]. However, the accumulation of adrenoceptor agonists in animal-derived foods presents a potential threat to the safety of consumers, frequently resulting in palpitations, muscle tremors, nausea, vomiting, and other clinical symptoms. These effects can be especially detrimental to individuals with heart disease, diabetes, and hypertension [2]. Traditional adrenoceptor agonists such as clenbuterol (CLE), salbutamol (SAL), dopamine (DA), and ractopamine (RAC) are commonly utilized [3]. In recent years, several new adrenoceptor agonists, including cyproheptadine (CYP), and clonidine (CLO), have been developed. China has implemented enhanced regulations for the control of adrenoceptor agonists in the feed and breeding sectors, leading to successful crackdowns on the illicit production, sale, and utilization of prohibited substances, thereby effectively mitigating their unlawful abuse. Nevertheless, certain farms persist in the use of adrenoceptor agonists driven by economic motivations. An incident concerning the illegal use of adrenoceptor agonists by sheep farmers was reported by CCTV in 2021. Therefore, it is urgent to improve the detection of adrenoceptor agonists, continually strengthen risk surveillance, identify potential risks associated with adrenoceptor agonists, and enhance the monitoring of animal-derived foods for safety purposes.

**Figure 1:**
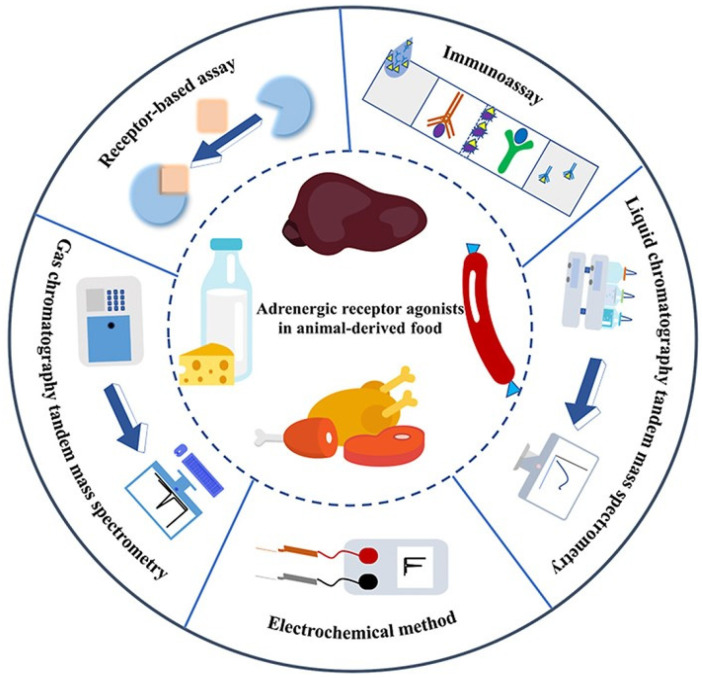
Methods for detecting β-adrenoceptor agonists [16] (reproduced with permission from Elsevier Ltd.). Reprinted/adapted with permission from Ref. [16]. 2024, 2024 Elsevier B.V.

**Figure 2:**
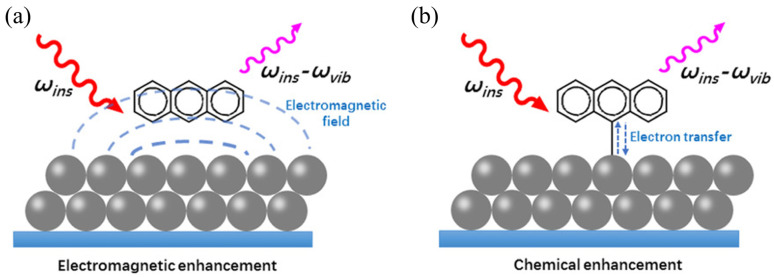
Schematic illustrations of electromagnetic (**a**) and chemical enhancement of Raman scattering signals of a molecule adsorbed on the surface of noble-metal nanoparticles (**b**) [13] (reproduced with permission from John Wiley and Sons). Reprinted/adapted with permission from Ref. [13]. 2024, 1999–2024 John Wiley & Sons, Inc. or related companies.

Typically, residues of adrenoceptor agonists in animal-derived food and animal urine are detected using analytical techniques such as gas chromatography/mass spectrometry (GC/MS) [4], liquid chromatography tandem mass spectrometry (LC-MS/MS) [5], immunoassays [6], electrochemical methods [7], and receptor-based assays, as demonstrated in Figure 1. Immunoassays are the primary approach for on-site monitoring of the use of illicit adrenoceptor agonists in animal agriculture [8]. Compared to these traditional methods, SERS is a technology developed from conventional Raman spectroscopy which utilizes Raman scattering to enable fast, nondestructive, and highly sensitive detection of adrenoceptor agonists. In the absence of signal enhancement, conventional Raman spectroscopy produces a low-intensity Raman signal which is not suitable for quantitative analysis. In contrast, SERS is able to produce stronger signals due to the presence of Raman reporter molecules and substrates. Further, the detection sensitivity and spatial discrimination have continuously been improved and extended, which further sets SERS apart from conventional Raman spectroscopy [9,10,11]. The literature generally reports two mechanisms to describe the effect of SERS: electromagnetic enhancement (EM) and chemical enhancement (CM) or electron transfer (Figure 2) [12,13]. However, SERS in dye molecules with conjugated systems can arise either from a general plasmonic enhancement resulting from the optical properties of the substrate or from a more analyte-specific chemical enhancement through molecule-substrate interactions [14]. It has been widely used in biosensing applications to achieve high analytical sensitivity [15].

Regarding the advantages of simple, fast, and sensitive detection, the developed SERS techniques are more adapted to practical applications when coupled with chemometric algorithms. SERS spectra can be affected by factors such as noise, baseline drift, particulate scattering, and changes in the refractive index, so multivariate calibration and pre-processing tools are required to process the spectral data [17]. In food matrices, chemometrics provides assistance in the analysis of complex components. In quantitative or qualitative analyses, chemometrics can be performed at different stages: (1) during data pre-processing, it can reduce interferences from particulate scattering, baseline drift, background noise, etc.; (2) selecting a calibration model based on pre-processed full spectral data; and (3) evaluating the performance of the constructed model through prediction accuracy and robustness [18]. For example, Guo et al. [19] collected SERS spectra of CLE in pork samples using gold colloid as an enhancement substrate. Multiple scattering correction (MSC) and automatic Whitaker filtering (AWF) were used for pre-processing to remove the fluorescence background contained in the original Raman spectra. The performance of the model was evaluated based on the correlation coefficient (R^2^) and root mean square error of correction (RMSEC). And the model of this study had better performance with good linearity (R^2^ = 0.99) and lower root mean square error (RMSEC = 0.263 μg/g). With the development of SERS and advances in chemometrics, the detection limits of adrenoceptor agonists will also be reduced [20]. Table 1 lists the use of SERS combined with chemometrics in the detection of adrenoceptor agonists.

SERS combined with chemometrics requires more complex data analysis and requires technicians for data processing [21]. It also lacks specificity and cannot quickly identify specific targets. As a result, SERS combined with immunoassays has emerged, which is able to specifically identify the target and requires much simpler data processing. SERS-IA is a novel labeling technique in which SERS-enhanced signals are utilized as a readout strategy. These methods are typically developed by capturing the analyte and subsequently modifying the immune substrate, followed by quantification of its concentration through the SERS immunoprobe [16]. In particular, the high sensitivity and spectral resolution of SERS are crucial for immunoassays. IA increases assay specificity by allowing specific antibodies to identify and bind to targets in complex molecular mixtures. The combination of SERS and IA enhances the stability and specificity of the result, enabling rapid trace detection due to the remarkable sensitivity of SERS. SERS-IA is widely used due to its advantages, such as high sensitivity, multicomponent detection, and good specificity, but it is still in the initial stages of the rapid detection of adrenoceptor agonists. SERS-IA comprises three components that are complementary and essential: Raman reporter molecules, substrate materials, and immunoreactivity. As immunoassay technology continues to mature and advance, researchers are dedicated to exploring substrate materials and Raman reporter molecules based on SERS-IA. Therefore, in this paper, we summarize the application of SERS-IA for the detection of rapid adrenoceptor agonists, along with commonly used substrate materials and Raman reporter molecules. This provides a reference for further research on the application of SERS-IA for the rapid detection of adrenoceptor agonists.

**Table 1:** Application of SERS combined with chemometrics in detecting adrenoceptor agonists.

Adrenoceptor Agonists	Characteristic Peaks (cm^−1^)	Data Processing Methods	Substrate	Excitation Wavelength	Laser Power	Integration Time	Reference
Clenbuterol	1259, 1472	AWF, MSC, RMSEC	Gold colloid	785 nm	100 mW	4 s	[19]
		partial least squares (PLS), binning, Gaussian smoothing, second-derivative transformation	/	780 nm	10mW	10 s	[22]
Ractopamine	830, 1000, 1168, 1192, 1262, 1498, 1588	Adaptive iteratively reweighted penalized least squares (AIR-PLS), wavelet transform, least squares support vector machine (LSSVM)	/	785 nm	200 mW	10 s	[23]
Dopamine	749, 795, 947, 1287	Artificial Neural Networks (ANNs), Partial Least Squares Regression (PLSR)	AuNPs	785 nm	17.5 mW	10 s	[24]
		Gaussian-09	AgNPs	532 nm	100 μW	200 ms	[25]
Salbutamol	621, 814, 1253, 1489, 1609	Savitzky-Golay (SG) 5-point smoothing filter, AIR-PLS	AgNPs	785 nm	180 mW	5 s	[26]
		SG second-derivative transformation, smoothing, polynomial subtraction	rGO/AuNPs	785 nm	50 mW	5 s	[27]

## 2. Raman Reporter Molecules

Many spurious peaks appear in the direct detection of adrenoceptor agonists by SERS, leading to challenges in substance identification and detection. To resolve this issue, the incorporation of Raman reporter molecules can provide different characteristic peaks and signal amplification, facilitating the differentiation of substances. Selecting appropriate Raman reporter molecules allows the introduction of additional characteristic peaks into the spectrum, thereby improving the differentiation between distinct substances [28]. Additionally, Raman reporter molecules exhibit high sensitivity and are detectable even at low concentrations. Thus, reliable signals can be obtained through the enhancement effect of Raman reporter molecules, even at very low levels of the target substance [29].

SERS nanoprobes are optical nanoprobes designed to attach noble-metal nanoparticles to particular Raman reporter molecules. Coupling SRES substrates with Raman reporter molecules with distinctive peaks is a crucial stage in the fabrication of SERS probes [30]. Dye molecules and thio-small molecules are the primary Raman reporter molecules utilized in the construction of SERS probes. Nitrogen contained in dye molecules or sulfur-containing molecules exhibits a strong binding affinity to Au and Ag, leading to clear and distinguishable Raman characteristic peaks. The characteristic peaks of different dyes and thio-small molecules are displayed in Table 2.

### 2.1. Thio-Small Molecules

**Figure 3:**
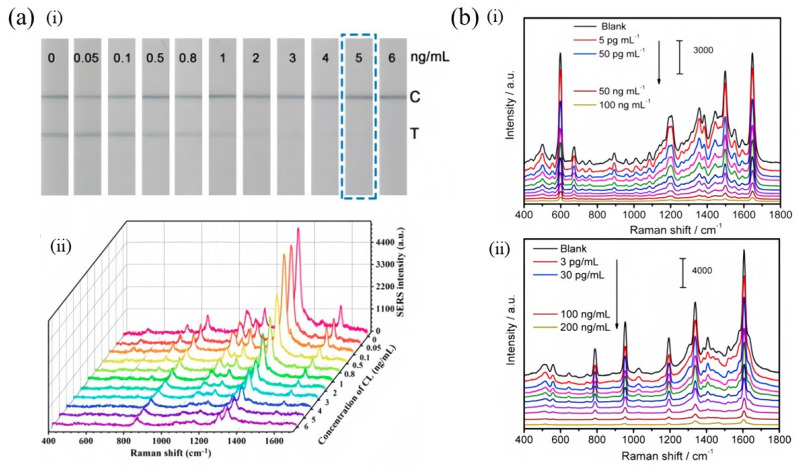
Photographs of test strips showing the colorimetric response at different CLE concentrations (**a**) (**i**); corresponding SERS spectra of DTNB within the test line at different CLE concentrations, the SERS signal decreased with the increase of CLE concentrations (**a**) (**ii**) [41] (open access from American Chemical Society). SERS spectra of NBs with increasing concentrations of SAL (**b**) (**i**); SERS spectra of TMB with increasing concentrations of BRO (**b**) (**ii**) [31] (reproduced with permission from Elsevier Ltd.). Reprinted/adapted with permission from Ref. [31]. 2024, 2024 Elsevier B.V.

Thio-small molecules are often used to enhance Raman signals because of their affordability, strong affinity for metal binding, and unique photoreactivity [39]. The interaction properties of thio-small molecules with gold nanoparticles (AuNPs) and silver nanoparticles (AgNPs) differ, where AuNPs exhibit a greater degree of direct interaction [40]. Yu et al. [38] described a highly sensitive method based on multiplex competitive SERS-IA. The first step involved the preparation of SERS nanoprobes. These nanoprobes were composed of AuNPs that were functionalized with 4,4′-dipyridyl (DP) and clenbuterol antibodies, as well as 2,2′-dipyridyl (BP) and ractopamine antibodies. In the second step, a competitive IA procedure was carried out. Standard solutions of CLE and RAC, along with equal volumes of the SERS nanoprobes, were applied to capture substrates that had been simultaneously immobilized with both CLE and RAC. Subsequently, the CLE and RAC present in the standard solutions could competitively bind with the CLE and RAC on the SERS nanoprobes, as well as those dropped onto the capture substrates. Finally, the capture substrates were irradiated using a microscopic Raman spectrometer, enabling the acquisition of SERS spectra of BP and DP to detect CLE and RAC with high sensitivity. This method achieved a detection limit of 1 pg/mL. Su et al. [41] developed a bimetallic core-shell Au/Au nanostar for the highly sensitive detection of CLE residues in food samples. To enhance the SERS signal, they incorporated 5,5′-dithiobis (2-nitrobenzoic acid) (DTNB), a Raman reporter molecule, between the core and shell of the nanostar. In the presence of the target analyte, the fixed complete antigen and free target analyte compete with the Au/Au nanostar signal probe. As the concentration of the target analyte increases, the detection signal weakens as a result of the competitive reaction. This change in signal intensity is reflected in the variation in color depth observed in the test line (T-line), allowing qualitative analysis using a colorimetric approach, as shown in Figure 3a(i). Furthermore, under excitation at 785 nm, the DTNB within the T-line exhibited different SERS spectra, as shown in Figure 3a(ii). Using excellent Raman reporter molecules, the competitive immunoassay for CLE had a detection limit of only 0.05 ng/mL according to SERS.

### 2.2. Dye Molecules

3,3′,5,5′-Tetramethylbenzidine (TMB) and Nile blue (NB) are dye molecules with rigid and planar structures, offering the advantages of low toxicity, low cost, and significant characteristic peaks [42]. In practical detection applications, dye molecules are not commonly used for the Raman detection of adrenoceptor agonists, as they are more frequently utilized for labeling cells and aptamers due to their bright, recognizable colors. Gu et al. [31] provided valuable insight into the synergistic use of multiple dye molecules to simultaneously detect multiple adrenoceptor agonists. They reported a competitive SERS-based IA developed for the detection of the β-adrenoceptor agonists SAL and brombuterol (BRO). The assay involved the preparation of highly ordered gold cavity arrays (GCAs), which exhibited an adjustable localized surface plasmon resonance and great Raman enhancement. It was achieved by electrodepositing gold nanoparticles onto polystyrene sphere template interstices and subsequently covering them with a prepunched sticker that functioned as detection areas. The immunoprobe was created by immobilizing antibodies against SAL (or BRO) and deoxyribonucleic acid (DNA) concatamers labeled with SERS reporters (NB and TMB) onto AuNPs. Following the immunoreaction, the characteristic peaks of NB and TMB captured in the GCAs were measured. The concentrations of SAL and BRO were determined and quantified by analyzing the characteristic wavelengths of the SERS peaks of NB and TMB, as shown in Figure 3b(i),(ii). Based on this modification, the group prepared a highly sensitive and multiplexed competitive immunoprobe for the simultaneous detection of the β-adrenoceptor agonists SAL, RAC, and phenylethanolamine A (PA). A highly ordered gold/silver bimetallic cavity array (BMCA) was prepared by electrodepositing Au/Ag NPs into the interstice of highly ordered closely packed PS templates. To serve as the dual-signal reporters, we selected TMB, MB, and NB, while a hybridization chain reaction (HCR) was used as a signal amplifier. The immunoprobe was prepared by absorbing the antibody and constructing an HCR system embedded with SERS reporters on AuNPs. After a competitive immunoreaction between the coating antigen and the analyte for limited antibodies on the immunoprobe, the SERS signals of TMB, MB, and NB were measured to allow for the simultaneous quantitative detection of SAL, RAC, and PA [33].

## 3. SERS Active Substrate

The materials used most frequently to produce active SERS substrates, which enhance SERS signals within the visible spectrum, are gold and silver [43]. While AgNPs offer superior SERS enhancements, AuNPs demonstrate greater stability, resilience against oxidation, and biocompatibility. Due to the intricate nature of the SERS phenomenon, diverse substrates are utilized to detect different analytes. Signal amplification is strongly influenced by the size, shape, and structure of active substrates [44]. With the development of nanosynthesis technology, a variety of metallic nanomaterials have been prepared as SERS active substrates. Currently, there are two main types of SERS active substrates: monometallic nanosubstrates and nanocomposite substrates. The detection sensitivity of nanocomposite substrates is greater than that of monometallic nanosubstrates [45]. Among nanocomposite substrates, core-shell structures have been most widely used, and Au@Ag and Ag@Au are the most classical bimetallic core-shell nanostructures. Fe_3_O_4_ is also used as a substrate material because of its excellent magnetic response properties [46].

### 3.1. Monometallic Nanosubstrates

The spherical colloidal gold nanoparticles commonly used in experiments can be synthesized by reducing metal salts using wet chemical methods [47]. The size and shape of nanoparticles have a significant impact on the properties of the metal substrate. When metal nanoparticles on SERS are too large, the Raman scattering enhancement decreases as a result of the occurrence of multipolar excitation. Studies suggest that nanoparticles ranging in size from 10 to 100 nm are much more effective in SERS experiments [48]. Guo et al. [49] conducted a comparative study on the adsorption efficiency of various AgNPs with different particle sizes in SAL. They discovered that larger AgNPs resulted in better enhancement of Raman signals. Therefore, it was concluded that larger AgNPs were more conducive to the adsorption of SAL. The LOD for SAL was estimated to be 0.2 mg/L under optimal conditions. When spherical nanoparticles undergo partial aggregation, the connections between the nanoparticles create SERS hotspots, leading to increased SERS efficiencies. However, techniques for precisely controlling nanoparticle aggregation are not yet well established, and consequently, the reproducibility of the SERS signal remains challenging [50].

**Figure 4:**
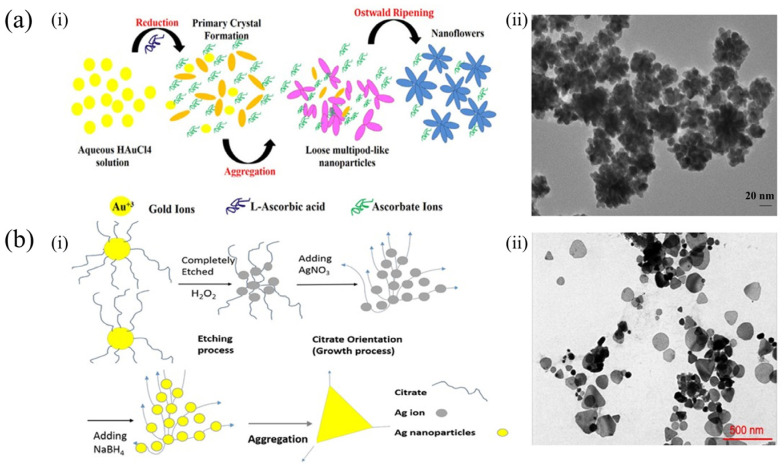
Synthesis of gold nanoflowers (**a**) (**i**); TEM image of AuNFs (**a**) (**ii**) [53] (reproduced with permission from Elsevier Ltd.). Synthesis of MAgNPs (**b**) (**i**); TEM images of MAgNPs (**b**) (**ii**) [54] (reproduced with permission from Springer Nature). Reprinted/adapted with permission from Ref. [53]. 2024, 2024 Elsevier B.V. Reprinted/adapted with permission from Ref. [54]. 2024, 2024 Springer Nature.

The shape of nanoparticles is one of the essential factors in enhancing the SERS performance of nanoparticle suspensions consisting of metals. Many shapes, including nanorods, nanoflowers, nanocubes, and nanostars, have been suggested and utilized in the generation of various biosensors [51]. To produce high-quality and adjustable homogeneous metal nanoparticles, researchers have attempted to optimize not only their size but also their shape through seed growth techniques. Fu et al. [52] reported that using gold nanoflowers (AuNFs) as a substrate could lead to a higher SERS signal. The unique design of the AuNFs generated many sharp branches, which resulted in the strongest SERS signal and boosted the sensitivity of the SERS analysis. Patel et al. [53] synthesized AuNFs with an average size of 45 nm. These particles exhibited a strongly enhanced electromagnetic field, which promoted the enhancement of Raman signals. Figure 4a(i) shows a schematic of the synthesis of AuNFs. Furthermore, the transmission electron microscopy (TEM) image of the AuNFs is presented in Figure 4a(ii). Moreover, Pham et al. [54] synthesized multi-shaped silver nanoparticles (MAgNPs) and compared them to silver nanospheres (AgNSs), as shown in Figure 4b(i). The MAgNPs exhibited various anisotropic shapes, such as hexagonal, circular, triangular, and deformed plates, together with particles in a wide range of sizes from 30 to 250 nm, shown in the TEM micrograph in Figure 4b(ii).

### 3.2. Nanocomposite Substrates

The use of monometallic NPs in SERS-IA for the analysis of complex samples still has limitations and needs urgent improvement. During trace analysis, the Raman reporter molecules adsorbed on monometallic nanosubstrates are susceptible to environmental interference, resulting in inaccurately detected signals [55]. In recent years, with the development of nanosynthesis technology, multicomponent nanostructures have become promising substrates for SERS [56]. Nanocomposite substrates are increasingly being investigated as SERS substrates due to their greater signal enhancement and better biocompatibility. These substrates are commonly employed to establish a protective layer that prevents nanoparticle aggregation and facilitates the adsorption of Raman reporter molecules onto metal substrates. Moreover, nanocomposite substrates have both metal-metal and metal-nonmetal compositions.

#### 3.2.1. Metal-Metal Substrates

**Figure 5:**
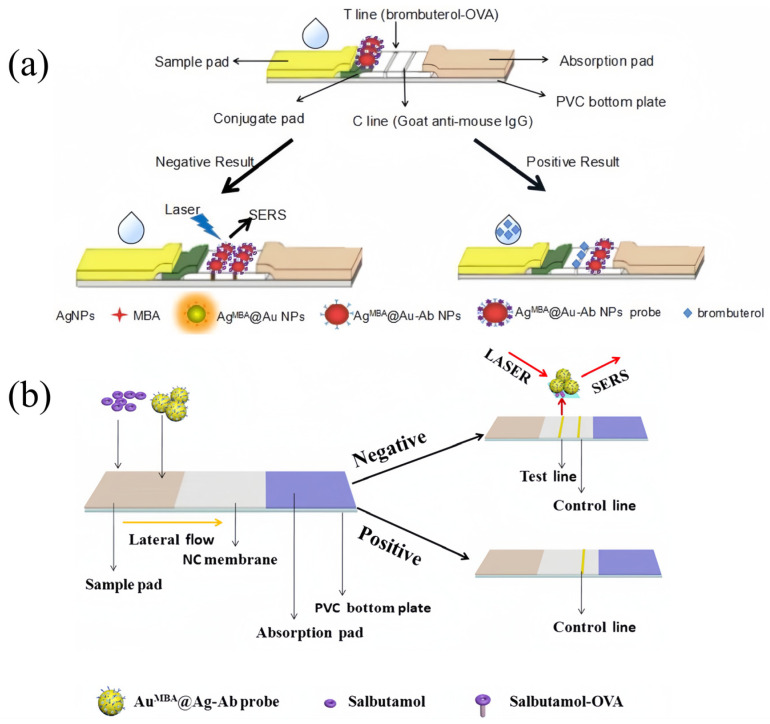
Assembly of the LFIA strip and the competitive SERS-LFIA principle for brombuterol detection (**a**) [35] (reproduced with permission from Royal Society of Chemistry); assembly of the LFIA strip and the SERS-LFIA scheme for SAL detection (**b**) [58] (reproduced with permission from Springer Nature). Reprinted/adapted with permission from Ref. [35]. 2024, The Royal Society of Chemistry 2021; Reprinted/adapted with permission from Ref. [58]. 2024, 2024 Springer Nature.

Core-shell Au/Ag nanoparticles are easy to synthesize, and the electronic ligand effect and local electric field enhancement of the core-shell nanostructures result in a higher SERS activity of bimetallic nanoparticles [57]. There are several studies on the use of bimetallic substrates for the detection of adrenoceptor agonists. For example, Huang et al. [35] created a Ag@Au core-shell for use as a substrate. To prepare the Ag^MBA^@Au-Ab immunoprobe, they enclosed thiosalicylic acid (MBA), the Raman reporter molecule, between the core and shell layers and immobilized a monoclonal antibody (mAb) against BRO on the surface of Ag^MBA^@Au. The embedded MBA possessed a strong electromagnetic field that significantly intensified its Raman signal. Additionally, the MBA was shielded by the Au shell from any degradation or leakage, and the MBA was protected from aggregation-induced enhancement. This method developed SERS-based immunochromatographic test strips, and the T-line and control line (C-line) were indeed formed by BRO-OVA and goat anti-mouse IgG, respectively. In a positive situation where the concentration of BRO is high, Ag^MBA^@Au-Ab on the conjugate pad binds primarily with BRO, leaving no free antibody binding sites for BRO-OVA. This prevents the immunoprobe from being captured on the T-line. Furthermore, quantitative detection of BRO was achieved by examining the characteristic Raman peak intensity of MBA in the immunoprobe captured by coating antigen on the T-line, as shown in Figure 5a. Similarly, Zhang et al. [58] employed MBA as a Raman reporter molecule and gold-silver core-shell bimetallic nanoparticles (Au^MBA^@Ag-Ab) as substrates to detect SAL in pork and urine samples. The use of core-shell nanoparticles prevented MBA from being desorbed, providing reliable quantitative detection. Following the procedures of the SERS-based lateral flow immunochromatographic assay (LFIA), a portable Raman spectrometer was used to obtain the SERS signal of MBA on the T-line, as shown in Figure 5b.

#### 3.2.2. Metal-Nonmetal Substrates

With the emergence of carbon materials, researchers have begun researching carbon composites as SERS substrates [59]. Yao et al. [60] prepared nitrogen/silver-codoped carbon dots (CD_N/Ag_) via highly catalytic amplification. They coupled CD_N/Ag_ catalytic amplification with the specific immunoreaction of CLE using highly sensitive SERS, resulting in a new spectroscopic strategy. CD_N/Ag_ nanoparticles can strongly catalyze the reaction between trisodium citrate and HAuCl_4_, leading to the generation of red-colored gold nanoparticles that exhibit a Raman signal. When CLE is present in the sample, a specific immune reaction occurs between Ab and CLE. This reaction causes the Ab to detach from the CD_N/Ag_ nanoparticles, restoring their catalytic activity and resulting in an increase in the Raman signal. The Raman signal is enhanced as the concentration of CLE increases.

Furthermore, Fe_3_O_4_ has garnered attention from researchers for its capacity to enrich targets through the utilization of its magnetic properties [61]. Wu et al. [62] developed an ultrasensitive immunochromatographic assay (ICA) using magnetic Fe_3_O_4_@Au-DTNB-Ab nanoprobes as a capture/detection difunctional tool for the detection of β_2_-adrenoceptor agonists in pork. In this method, goat anti-mouse antibody, RAC-BSA, and CLE-BSA are evenly sprayed onto nitrocellulose (NC) membranes to form the C-line, T_1_-line, and T_2_-line, respectively. The principle of competition is utilized, where the target analytes in the sample solution compete with the antigen immobilized on the T-line to bind to Fe_3_O_4_@Au-DTNB-Ab. The target analytes CLE/RAC are captured by Fe_3_O_4_@Au-DTNB-Ab and subsequently enriched using a magnetic field. The quantitative detection of CLE/RAC was achieved by measuring the intensity of the Raman signal produced by DTNB. This study employs the distinctive magnetic response properties of magnetic materials to enrich targets in complex environments while mitigating interference from other matrices, thereby demonstrating significant practical applications.

The development of SERS substrates with the advantages of low cost, simple fabrication, good flexibility, and high sensitivity has been the focus of SERS analysis in the food industry [63]. Many efforts have been made in the development of flexible substrate materials, and flexible SERS substrates have been developed for the real-time and in situ detection of irregular surfaces [64]. Viriyakitpattana et al. [65] demonstrated a simple and cost-effective plasmonic paper by the in situ synthesis of AuNPs on filter paper. The plasmon coupling of high-density AuNPs on the substrate improved the signal enhancement. When utilizing SERS on plasmonic paper, the liquid sample was simply loaded via capillary force by dipping the plasmonic paper into the sample. The paper was used as a microfluidic for liquid sample transportation, concentrating the analytes into specific areas through solvent evaporation, producing a strong SERS signal. The paper-based SERS substrate developed in this study was able to simultaneously detect two types of β-agonists, RAC and SAL in solution, demonstrating the multiplexing capability and versatility of the plasmonic paper in food contaminant analysis.

## 4. Summary and Further Perspectives

This review provides a summary of the categorization of substrate structures and Raman reporter molecules used in SERS-IA, along with an overview of recent research on SERS-IA for adrenoceptor agonists. Table 3 displays the application of SERS-IA in detecting adrenoceptor agonists. It is evident that many methods still use classic metal substrates, with fewer incorporating nonmetal substrates. In fact, nonmetal substrate materials also have high practical application value. For example, Fe_3_O_4_ can not only enhance the Raman effect but also utilize magnetism to enrich the targets, greatly improving the sensitivity and efficiency of SERS-IA [66]. In addition to traditional substrates, flexible substrates have attracted more and more attention from researchers. The advantages of using flexible SERS substrates are significant. Flexible SERS substrates can be cut into the desired shape and size for on-demand use; they can be easily swabbed or wrapped on sample surfaces, enabling nondestructive and in situ detection [67]. More importantly, wearable and light-weight flexible SERS substrates can be combined with portable Raman devices or even with smartphones to provide possible on-site detection, which can be used as a dynamic analytical tool in real-world applications for food safety analysis [68].

Raman reporter molecules play a crucial role in creating nanogaps within nanoparticles, thereby generating “hot spots” that significantly enhance SERS activity. However, despite their extensive use, these Raman reporter molecules face significant challenges such as poor SERS activity, the occurrence of hot spots, and instability when dealing with complex samples. To ensure the successful detection of adrenoceptor agonists, it is imperative to achieve improved analytical sensitivity, especially given the complexity of food matrices. Therefore, producing ultracompact Raman reporter molecules that offer improved sensitivity and reproducibility becomes essential.

Compared to other immunoassay methods, SERS-IA requires a greater quantity of antibodies to construct SERS nanoprobes. Therefore, it is necessary to design substrate structures in a rational manner to improve the efficiency of antibody binding and minimize the amount of antibodies used. Additionally, uncontrolled nanoparticle aggregation may induce the generation of “hot spots”, leading to substantial alterations in SERS signals and nonspecific adsorption of immune probes. Therefore, it is essential to make further efforts to achieve a balance between the stability of SERS immune probes and high SERS activity.

Although SERS-IA is able to specifically recognize the targets, the samples need to be pre-treated. The future analytical techniques emphasize the application of nondestructive testing techniques combined with chemometrics to process Raman signals, enabling in situ, rapid, accurate, and high-throughput analysis. Furthermore, integrating SERS technology with other techniques, such as chemical separation and biological capture technologies, can enhance sensitivity. In order to obtain results quickly and conveniently, the development of portable Raman spectrometers holds significant promise for rapid detection applications [69]. The continuous development of nanotechnology has raised the feasibility of miniaturized Raman spectrometers, but achieving high resolution to distinguish multipeak features in complex Raman spectra is also a major challenge for miniaturized Raman spectrometers [70]. In the increasing evolution of miniaturized Raman spectrometers, it has been found that single-band Raman analysis enables highly stable and accurate biosensing, so it is beginning to be a hotspot for biosensing research in miniaturized Raman spectrometers [71,72]. The development of smaller, higher-performance, and higher-sensitivity Raman spectrometers will be a trend [73], and the integration of miniaturized Raman spectrometers enables SERS to be an effective analytical tool in the development of portable and automated detection platforms [74]. The continuous development of portable Raman spectrometers can provide a new method for on-site and real-time testing in different fields.

In conclusion, SERS-IA has tremendous potential for detecting adrenoceptor agonists in food; the continuous development of Raman systems, nanomaterials, and spectral analysis algorithms will facilitate the transfer from laboratory to industry [75]. Ongoing research should prioritize the design of multifunctional substrates and nanoprobes and the development of portable and efficient analysis platforms to advance the progress and application of this field.

**Table 3:** Application of SERS-IA in detecting adrenoceptor agonists.

Substrate	Raman Reporter	Excitation Wavelength	Laser Power	Integration Time	Target	Sample	LOD	Reference
Au cavity arrays	NB, TMB	638 nm	0.5 mW	10 s	SAL, BRO	Pork meat, pork liver and human urine	2.0, 1.0 pg/mL	[31]
Au/Ag bimetallic cavity array	MB	638 nm	0.6 mW	/	SAL, RAC and PA	Pork liver	0.8, 0.4, and 1.3 pg/mL	[33]
AuNPs	Prussian blue (PB)	785 nm	10 mW	10 s	RAC	RAC standard solution	0.08 ng/mL	[76]
Fe_3_O_4_@Au	DTNB	785 nm	10 mW	10 s	CLE, RAC	Pork, mutton, and beef	7.8, 3.5 pg/mL	[62]
Au@Ag	MBA	785 nm	20 mW	10 s	PA	Pork	0.32 pg/mL	[77]
Ag@Au	MBA	785 nm	50 mW	10 s	BRO	BRO standard solution	0.11 pg/mL	[35]
AuNPs	DP, BP	632.8 nm	1 mW	30 s	CLE, RAC	CLE and RAC standard solutions	1.0, 1.0 pg/mL	[38]

## Figures and Tables

**Table 2:** Typical types of Raman reporter molecules for SERS detection of adrenoceptor agonists.

Type	Raman Reporter Molecules	Characteristic Peaks (cm^−1^)	Reference
Dye molecules	Nile blue (NB)	592, 1638	[31]
	Rhodamine 6G (R6G)	774, 1510	[32]
	Methylene Blue (MB)	445, 664	[33]
	Malachite green isothiocyanate (MGITC)	210, 419	[34]
Thio-small molecules	4-mercaptobenzoic acid (4-MBA)	1077, 1587	[35]
	4-aminothiophenol (4-ATP)	1079, 1590	[36]
	5,5-dithiobis-2-nitrobenzoic acid (DTNB)	1331	[37]
	4,4′-bipyridyl (DP)	999, 1297	[38]

## Data Availability

No new data were created or analyzed in this study. Data sharing is not applicable to this article.
